# Comparison of the Effects of Phenylhydrazine Hydrochloride and Dicyandiamide on Ammonia-Oxidizing Bacteria and Archaea in Andosols

**DOI:** 10.3389/fmicb.2017.02226

**Published:** 2017-11-14

**Authors:** Wenjie Yang, Yong Wang, Kanako Tago, Shinichi Tokuda, Masahito Hayatsu

**Affiliations:** ^1^Jiangsu Key Laboratory for Eco-Agricultural Biotechnology around Hongze Lake, Huaiyin Normal University, Huai’an, China; ^2^Division of Biogeochemical Cycles, Institute for Agro-Environmental Sciences, National Agriculture and Food Research Organization, Tsukuba, Japan; ^3^Western Region Agricultural Research Center, National Agriculture and Food Research Organization, Kyoto, Japan

**Keywords:** nitrification inhibitor, ammonia-oxidizing bacteria, ammonia-oxidizing archaea, Andosol, phenylhydrazine hydrochloride, dicyandiamide

## Abstract

Dicyandiamide, a routinely used commercial nitrification inhibitor (NI), inhibits ammonia oxidation catalyzed by ammonia monooxygenase (AMO). Phenylhydrazine hydrochloride has shown considerable potential for the development of next-generation NIs targeting hydroxylamine dehydrogenase (HAO). The effects of the AMO inhibitor and the HAO inhibitor on ammonia-oxidizing bacteria (AOB) and ammonia-oxidizing archaea (AOA) present in agricultural soils have not been compared thus far. In the present study, the effects of the two inhibitors on soil nitrification and the abundance of AOA and AOB as well as their community structure were investigated in a soil microcosm using quantitative polymerase chain reaction and pyrosequencing. The net nitrification rates and the growth of AOA and AOB in this soil microcosm were inhibited by both NIs. Both NIs had limited effect on the community structure of AOB and no effect on that of AOA in this soil microcosm. The effects of phenylhydrazine hydrochloride were similar to those of dicyandiamide. These results indicated that organohydrazine-based NIs have potential for the development of next-generation NIs targeting HAO in the future.

## Introduction

Nitrification, the process of microbial oxidation of ammonia to nitrate (NO_3_^-^), is essential in the global nitrogen (N) cycle. In agricultural ecosystems, rapid and unregulated nitrification results in a range of issues such as inefficient N use by crops, loss of N from agricultural ecosystems through leaching of ${\mathrm{NO}}_{3}^{–}$, nitrous oxide (N_2_O) emission, and environmental pollution (Likens et al., 1969; Schlesinger, 2009). Generally, in the first step of nitrification, ammonia is oxidized to hydroxylamine (NH_2_OH) by ammonia monooxygenase (AMO, EC 1.14.99.39) and further to nitrite (${\mathrm{NO}}_{2}^{–}$) by hydroxylamine dehydrogenase (formerly hydroxylamine oxidoreductase) (HAO, EC 1.7.2.6) (Arp and Stein, 2003). Oxidation of ammonia, the first and rate-limiting step in nitrification, is carried out by both ammonia-oxidizing bacteria (AOB) and ammonia-oxidizing archaea (AOA) (Leininger et al., 2006; He et al., 2007). Therefore, suppression of AOA and AOB growth effectively reduces N loss from the soil as well as pollution.

The addition of nitrification inhibitors (NIs) is a promising technique for reducing the amount of N lost through leaching and the amount of N_2_O produced directly by ammonia oxidizers in soil. It also reduces N_2_O emission in agricultural ecosystems by increasing N retention time in the root zone in the form of ${\mathrm{NH}}_{4}^{+}$ (Cui et al., 2011), providing additional time for the plants to absorb N (Hodge et al., 2000; Subbarao et al., 2012). Moreover, the use of NIs is an effective technique for mitigation of N_2_O emissions as well as ${\mathrm{NO}}_{3}^{–}$ leaching (Akiyama et al., 2010). The mode of action of NIs has been reviewed (McCarty, 1999), and approximately 60 compounds were reported to influence AMO activity by acting as its alternative substrates (Subbarao et al., 2006). A previous study shows that dicyandiamide (DCD), the most commonly used commercial NI, inhibits AMO activity, probably by impairing the uptake or utilization of ammonia (Zacherl and Amberger, 1990). In the past decades, because of its high efficacy in inhibiting nitrification, DCD has been used extensively as an NI and has gained practical and commercial importance in agricultural production (de Klein et al., 2001; Di and Cameron, 2002, 2006, 2007; de Klein and Eckard, 2008; Smith et al., 2008; Di et al., 2009, 2010b).

Previous studies suggested that chemical and physical properties of soil and agricultural management strategies account for considerable differences in the abundance, activity, and community structures of AOB and AOA because of the differences in their cellular biochemistry and physiology (He et al., 2007; Nicol et al., 2008; Jia and Conrad, 2009; Gubry-Rangin et al., 2011; Morimoto et al., 2011; Verhamme et al., 2011; Xia et al., 2011; Yao et al., 2011; Shen et al., 2012; Tago et al., 2015). Moreover, AOA and AOB show different susceptibilities to DCD. It was reported that the community of AOB shifted in response to DCD addition, whereas that of AOA showed no response (O’Callaghan et al., 2010). Shen et al. (2013) reported a markedly lower effect of DCD on ammonia oxidation in pure cultures of AOA than that observed in those of AOB. Similarly, the copy number of AOB *amoA* was considerably reduced compared to that of AOA *amoA* after the addition of DCD in the presence of high N concentrations (Patra et al., 2006; Singh et al., 2008; Carneiro et al., 2010; O’Callaghan et al., 2010; Dai et al., 2013). In another study, the addition of DCD not only significantly inhibited the nitrification but also decreased the AOB abundance and altered the AOB community (Liu et al., 2014). However, to date, there have been few reports examining the effects of DCD on the nitrification, abundance, and community of AOA and AOB simultaneously, which are required to better understand the inhibition of nitrification by DCD.

Alkyl- and aryl-hydrazine derivatives are potential NIs, which can irreversibly inactivate HAO by covalent modification in the active site of the enzyme (Logan and Hooper, 1995). HAO is the key enzyme for conversion of hydroxylamine to ${\mathrm{NO}}_{2}^{–}$ and is involved in producing energy to support the growth of AOB during active nitrification (Arp et al., 2007). There are few reports on the effects of organohydrazines on soil ammonia oxidizers and on their inhibitory effect on bacterial HAO, which was initially observed in cell-free enzyme extracts (Logan and Hooper, 1995). Kane and Williamson (1983) reported that the N metabolism of *Nitrosomonas* was inhibited effectively *in vivo* by methylhydrazine. Previous studies suggested that no bacterial HAO gene (*hao*) homolog was present in the genome of AOA isolates or in the enrichment cultures (Hallam et al., 2006; Walker et al., 2010; Blainey et al., 2011). Thus, if nitrification is inhibited because of the inactivation of HAO, the nitrification activity of AOA should not be affected. A recent report revealed that soil nitrification was completely suppressed by high concentration of organohydrazines (100 mmol⋅kg^-1^ soil) and correlated well with the AOB rather than AOA community structure (Wu et al., 2012). Thus, organohydrazines have considerable potential for the development of next-generation NIs targeting HAO (Nishigaya et al., 2016), even though most commercially available NIs target AMO. Most recently, Nishigaya et al. (2016) proposed the development of NIs targeting HAO such as organohydrazine based on the structure-guided drug design using the information from 3D structures of the target molecule and the target-ligand complex for drug discovery. However, to date, very limited information about the distinct effects of organohydrazines on ammonia oxidizers in agricultural soils is available.

The objective of the present study was to compare the effects of phenylhydrazine hydrochloride (PHH), an HAO inhibitor, and DCD on the nitrification activity, abundance, and community structure of AOA and AOB simultaneously in agricultural Andosols, using quantitative polymerase chain reaction (qPCR) and pyrosequencing techniques.

## Materials and Methods

### Soil Incubation

A soil sample (pH 5.4) was obtained from an agricultural field at the Institute of Vegetable and Tea Science, National Agriculture and Food Research Organization (NARO) (36.016° N, 140.108° E) in Tsukuba, Ibaraki prefecture, Japan, and was homogenized and incubated in the dark for 3 days at 25°C. Before further analysis and incubation, the maximum water-holding capacity (WHC) of the field-fresh soil was determined. The moisture content of the fresh soil was 32.79% and the maximum WHC was 49.25%.

In a preliminary soil incubation experiment, the soil was treated with different concentrations of DCD or PHH (Tokyo Chemical Industry Co., Ltd, Tokyo, Japan) for 7 days at 25°C in the presence of ammonium sulfate [(NH_4_)_2_SO_4_] (280 mg⋅kg^-1^ dry soil) at a level normally applied in cabbage fields. The levels of ${\mathrm{NO}}_{3}^{–}$ in the soil at days 0 and 7 were measured; based on these levels, the optimal concentration of DCD and PHH for inhibiting the ammonia-oxidizing activities was determined.

We set up the following three treatments (in triplicate): soil amended with 280 mg ammonium nitrogen (${\mathrm{NH}}_{4}^{+}$–N) kg^-1^ dry soil (Control), soil amended with 280 mg ${\mathrm{NH}}_{4}^{+}$–N kg^-1^ dry soil + 10 mmol⋅kg^-1^ dry soil PHH (PHH), and soil amended with 280 mg ${\mathrm{NH}}_{4}^{+}$–N kg^-1^ dry soil + 5 mmol⋅kg^-1^ dry soil DCD (DCD). (NH_4_)_2_SO_4_ was used as ${\mathrm{NH}}_{4}^{+}$ source. For each treatment, 42.25 g fresh soil (30 g dry soil) was added to a 100-mL loosely capped flask and thoroughly mixed with (NH_4_)_2_SO_4_ and 10 mmol⋅kg^-1^ dry soil PHH or 5 mmol⋅kg^-1^ dry soil DCD, which were dissolved in deionized water before addition to the soil. The soil moisture was adjusted to 60% of the maximum WHC. Microcosms of each treatment were incubated in the dark at 25°C for 14 days. Soil water content was monitored by weight and kept constant at 60% of the maximum WHC by addition of deionized water periodically. Triplicate microcosms were destructively sampled at 0, 3, 6, 10, and 14 days of incubation. Soil used for extraction and analysis of DNA was immediately stored at -20°C. The remaining soil of each microcosm was used for extraction and determination of inorganic N. The net nitrification rate (n) in the first 6 days (because ${\mathrm{NH}}_{4}^{+}$–N was consumed within 6 days in the Control soil) was calculated according to the equation developed by Persson and Wirén (1995).

### N Content Measurement

Briefly, 10 mL of 0.5 M K_2_SO_4_ solution was added to a 2-g soil sample, followed by shaking at 25°C for 30 min at 120 rpm and centrifugation at 13,000 × *g* for 10 min. The supernatant was transferred into a sterilized microcentrifuge tube for the measurement of inorganic N. ${\mathrm{NO}}_{3}^{–}$ was analyzed using the Cu-cadmium (Cd) reduction method, whereas ${\mathrm{NH}}_{4}^{+}$ was analyzed by the indophenol blue method (Rhine et al., 1998) in a continuous flow analyzer (TRAACS, Bran + Luebbe, Norderstedt, Germany).

### Quantitative Polymerase Chain Reaction (qPCR) for *amoA*

Total DNA was extracted from 0.4 g of soil of each sample using the FastDNA Spin kit for soil (Qbiogene, Inc., Irvine, CA, United States), according to the manufacturer’s instructions. Andosol contains high level of humic acids; therefore, further purification was performed to remove humic acids completely using MicroSpin S-400 HR columns (GE Healthcare, Little Chalfont, United Kingdom) and the DNA Clean and Concentrator-25 kit (Zymo Research, Irvine, CA, United States). The purified soil DNA, with concentrations ranging from 100 to 200 ng μl^-1^, as quantified using a NanoDrop 1000 spectrophotometer (Thermo Fisher Scientific, Waltham, MA, United States), was diluted 10-fold, and stored at -20°C until use.

Quantification of archaeal and bacterial *amoA* from total DNA extracts was performed on a StepOnePlus Real-Time PCR System (Applied Biosystems, Foster City, CA, United States) with SYBR Premix Ex Taq (TaKaRa Bio Inc., Shiga, Japan), using the primer set *amoA*19IF and *amoA*-616R (Tourna et al., 2008) for AOA, and *amoA*-1F (Stephen et al., 1998) and *amoA*-2R-GG (Nicolaisen and Ramsing, 2002) for AOB. The quantification of AOA *amoA* was performed in a 20-μL reaction volume consisting of 10 μL of SYBR Premix Ex Taq, 4 μg of bovine serum albumin, 200 nM of each primer, and 2 μL of 10-fold diluted DNA. The reaction system used for AOB was the same as that used for AOA except for the primer concentration (80 nM). The amplification conditions for AOA were as follows: an initial denaturation step at 94°C for 2 min, followed by 40 cycles of 30 s at 94°C, 30 s at 55°C, and 1 min at 72°C. The cycle conditions for AOB were the same except for the annealing temperature (54°C). Standard curves were generated using serial dilutions of linearized pGEM-T Easy plasmids (Promega, Fitchburg, WI, United States) containing cloned *amoA* [correlation coefficient (*R*^2^) > 0.99]. The efficiency of the qPCR reactions was 92% for AOA and 96% for AOB, and the calibration range was 10 to 1,000,000 copies for both. We did not examine the detection limit in our qPCR experiment. In theory, the detection limit of qPCR is three copies (Bustin et al., 2009), and the lower limit of the calibration range in these qPCR reactions was 10 copies; therefore, we believe that the detection limit of these qPCR reactions should be between 3 and 10 copies.

### Pyrosequencing Analysis of *amoA*

A total of 36 soil DNA samples (3 biological replicates × 2 time points × 3 treatments × 2 types of nitrifier), namely, for either AOA or AOB *amoA*, triplicated Control-0 day, Control-14 days, PHH-0 day, PHH-14 days, DCD-0 day, and DCD-14 days, were utilized for the 454-pyrosequencing analysis. To prepare the templates for pyrosequencing, PCR amplification was performed using fusion primers, which contained the sequences of adaptor, key, and multiplex identifier (MID) as well as the gene specific sequence. After purification using the QIAquick PCR Purification kit (Qiagen, Valencia, CA, United States), the PCR products were subjected to agarose gel electrophoresis, and the target DNA fragments in the gel were purified using the QIAquick Gel Extraction kit (Qiagen). The quality and quantity of purified DNA fragments were determined using an Agilent 2100 Bioanalyzer (Agilent Technologies, Santa Clara, CA, United States) and the Quant-iT PicoGreen dsDNA Assay kit (Life Technologies, Carlsbad, CA, United States), respectively. Pyrosequencing was carried out using the Roche 454 GS Junior sequencer (Roche Diagnostics, Basel, Switzerland) according to the 454 Life Sciences protocol (Roche Diagnostics).

### Sequence Analysis and Phylogenetic Assignment

Sequence analysis and phylogenetic assignment were performed as previously described (Tago et al., 2015). Briefly, the low quality reads in the raw pyrosequencing data were screened and eliminated using Mothur (Schloss et al., 2009). High quality sequences (no ambiguous base, average quality score higher than 25, and minimum sequence length of 425 nt for AOA *amoA* or 450 nt for AOB *amoA*) were analyzed further. The chimeras of the unique sequences were identified and removed using the UCHIME algorithm in Mothur. The processed sequences were submitted to the FrameBot (Wang et al., 2013)^1^ to correct sequencing errors resulting in frameshift. The sequences were clustered into operational taxonomic units (OTUs) with a cut-off value of 0.07 for AOA *amoA* and 0.05 for AOB *amoA* as previously described (Tago et al., 2015). The representative nucleotide sequences of each OTU were picked for phylogenetic analysis, which was performed using molecular evolutionary genetics analysis (MEGA) 6 software (Tamura et al., 2013). Reference sequences of *amoA* genes were obtained from NCBI. The sequences were aligned with the ClustalW program (Chenna et al., 2003). Each of the AOA- and AOB-*amoA* phylogenic trees was constructed by using the maximum likelihood method with the Jukes–Cantor model and assessed by using 1000 bootstrap replicates.

### Data Analysis

The differences in the number of *amoA* copies in AOA and AOB at days 0 and 14 were compared by a paired *t*-test using R^2^. Analysis of similarities (ANOSIM) and weighted and unweighted UniFrac were performed using Mothur. In addition, Metastats in Mothur was used to identify the OTUs showing significant variation among the different samples. Beta-diversities were presented as non-metric multidimensional scaling (nMDS) plots, which were generated from Hellinger-transformed Bray–Curtis matrices using the Vegan package in R.

### Accession Number of Nucleotide Sequences

All sequences acquired from pyrosequencing have been deposited to the DDBJ Sequence Read Archive under the accession number DRA005215.

## Results

### ${\mathrm{NH}}_{4}^{+}$ and ${\mathrm{NO}}_{3}^{–}$ Content in the Soil

According to the data acquired in the preliminary soil incubation experiment (Supplementary Figure S1), the optimal concentrations of DCD and PHH were 5 mmol⋅kg^-1^ dry soil and 10 mmol⋅kg^-1^ dry soil, respectively. Thus, we used these concentrations in the soil incubation experiment.

**FIGURE 1:**
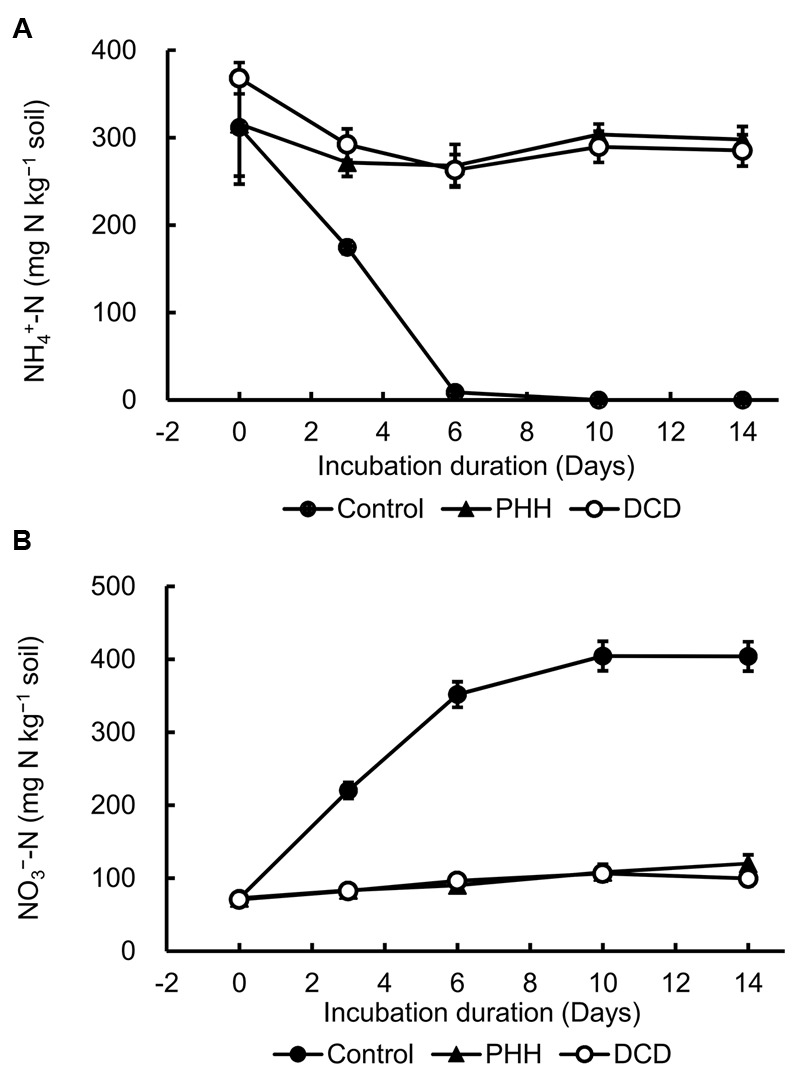
Dynamics of ammonium nitrogen (${\mathrm{NH}}_{4}^{+}$–N) **(A)** and nitrate nitrogen (${\mathrm{NO}}_{3}^{–}$–N) concentrations **(B)** in soil. Control, soil treated with ammonium sulfate [(NH_4_)_2_SO_4_]; phenylhydrazine hydrochloride (PHH), soils treated with (NH_4_)_2_SO_4_ + PHH; dicyandiamide (DCD), (NH_4_)_2_SO_4_ + DCD. Error bars represent the standard deviation of means (SD) (*n* = 3).

In the soil incubation experiment, the Control showed the highest ${\mathrm{NH}}_{4}^{+}$–N concentration at day 0, which declined quickly and reached a value of approximately 0 at day 10 (**Figure 1A**). The ${\mathrm{NH}}_{4}^{+}$–N concentrations in the N [(NH_4_)_2_SO_4_] + NIs (DCD or PHH) treatments decreased slightly in the first 3 days and then remained constant until the end of the soil incubation (**Figure 1A**).

As shown in **Figure 1B**, the highest concentration of nitrate nitrogen (${\mathrm{NO}}_{3}^{–}$–N) was detected at day 10 in Control and was maintained constant until the end of incubation. The ${\mathrm{NO}}_{3}^{–}$–N concentration in the soil with N + NIs (DCD or PHH) slightly increased during the entire incubation period (**Figure 1B**). These results were in agreement with the ${\mathrm{NH}}_{4}^{+}$–N concentrations. The net nitrification rates in Control, PHH, and DCD were 46.50 ± 0.42, 2.93 ± 0.10, and 4.22 ± 0.31 mg kg^-1^ day^-1^, respectively. Thus, PHH or DCD addition decreased the net nitrification rate by 14.8 and 10 times, respectively, compared to the rate in Control during the 0–6 day interval.

### AOA and AOB Abundance

**FIGURE 2:**
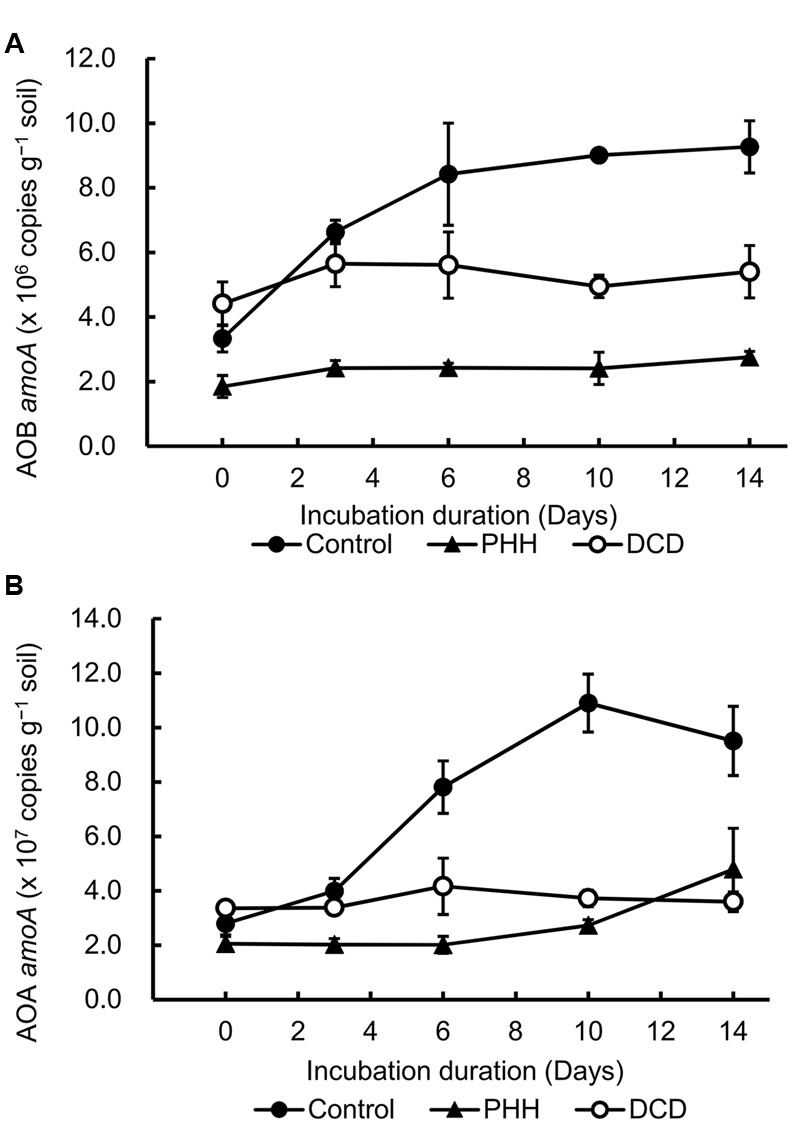
Ammonia-oxidizing bacteria (AOB) *amoA* copies **(A)** and ammonia-oxidizing archaea (AOA) *amoA* copies **(B)** detected by quantitative polymerase chain reaction (qPCR). Control, soil treated with ammonium sulfate [(NH_4_)_2_SO_4_]; PHH, soils treated with (NH_4_)_2_SO_4_ + PHH; DCD, (NH_4_)_2_SO_4_ + DCD. Error bars represent the standard deviation of means (SD) (*n* = 3).

The changes in the bacterial and archaeal *amoA* copy number, which reflected the abundance of AOB and AOA populations, were quantified using qPCR (**Figure 2**). The ratio of the number of copies in AOA and AOB ranged from 6.0 to 17.3. These results revealed that the AOA population size was much larger than that of AOB in the Andosols.

Ammonia-oxidizing bacteria *amoA* copy number in the Control samples increased significantly after incubation for 14 days (*p* = 0.004 in paired *t*-tests). However, in the PHH and DCD treatments, the AOB *amoA* copies did not show significant increase (*p* = 0.069 for PHH treatment; *p* = 0.211 for DCD treatment in paired *t*-tests) (**Figure 2A**). These data suggested that the growth of AOB was completely inhibited by PHH or DCD. As the result of inhibition, the AOB *amoA* copy numbers in the PHH and DCD treatments were markedly lower than those in the Control treatment after incubation for 14 days.

Similarly, addition of (NH_4_)_2_SO_4_ stimulated a significant increase in AOA *amoA* copy number in Control after incubation for 14 days (*p* = 0.014 in a paired *t*-test), whereas the AOA *amoA* copies in the PHH- and DCD-treated samples did not show significant increase (*p* = 0.115 for PHH treatment; *p* = 0.286 for DCD treatment in paired *t*-tests) (**Figure 2B**). Compared to Control, the AOA *amoA* copy number in the PHH and DCD treatments remained at low levels throughout the incubation period (**Figure 2B**) because of the inhibition effect of PHH or DCD. Addition of PHH and DCD not only inhibited the growth of AOB but also the growth of AOA.

### Phylogeny of AOA and AOB

Variation in AOB and AOA community structures was assessed by 454-pyrosequencing analysis of *amoA* in three treatments (Control, PHH, and DCD) at days 0 and 14. A total of 91,360 AOB and 92,975 AOA *amoA* sequence reads were obtained after excluding chimeric and low-quality sequences (Supplementary Table S1). The number of sequence reads in each sample ranged from 1,825 to 9,042 for AOB *amoA* and from 3,719 to 7,772 for AOA *amoA*. Rarefaction analysis revealed that all treatments were sampled almost to saturation in both AOB and AOA *amoA* analyses (library coverage > 97 and 99%, respectively).

**FIGURE 3:**
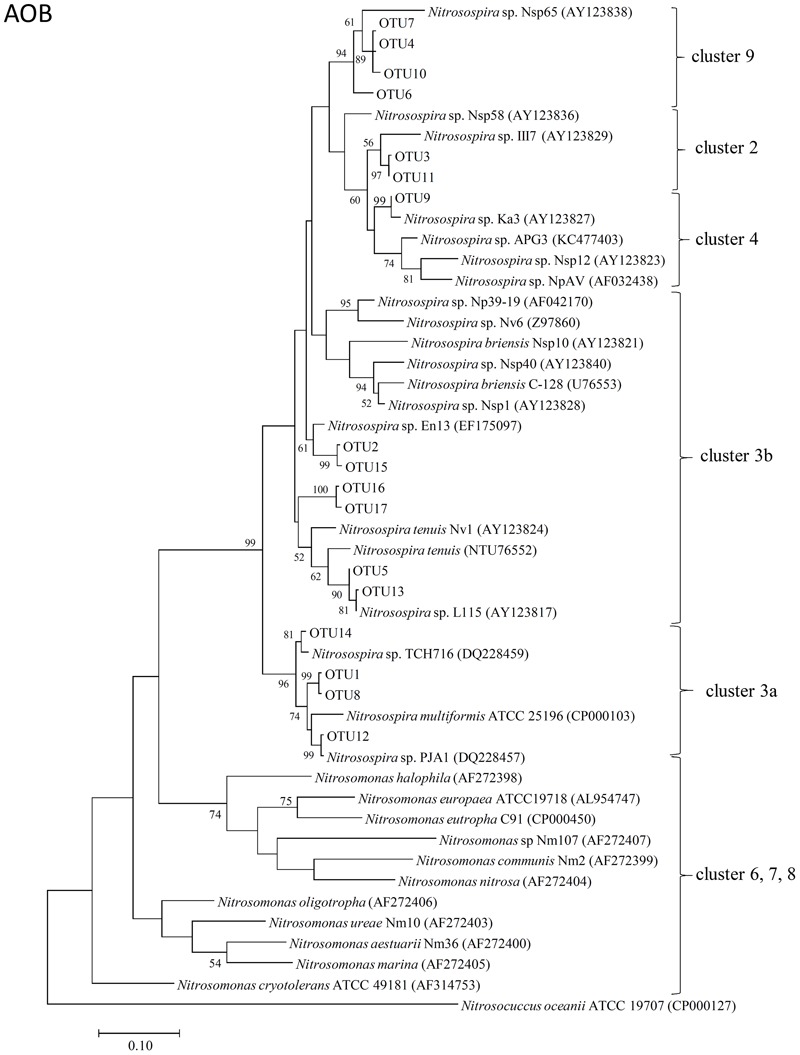
Neighbor-joining phylogenetic tree of bacterial *amoA* operational taxonomic units (OTUs) obtained in the present study. Bootstrap values (>50%) are indicated at branch points. The scale bar represents 10% estimated sequence divergence.

The representative sequences of 17 dominant OTUs (>1.0% in all samples) of AOB *amoA* were used for the phylogenetic analysis. The cluster identification of AOB *amoA* previously defined (Avrahami and Conrad, 2003, 2005; Avrahami et al., 2003; Zhang et al., 2009) was used for the analysis. All the AOB *amoA* OTUs detected in the soil samples were affiliated exclusively with the genus *Nitrosospira*, and 17 dominant OTUs were classified into five different clusters. There were four OTUs (OTU1, OTU8, OTU12, and OTU14) belonging to cluster 3a, six OTUs (OTU2, OTU5, OTU13, OTU15, OTU16, and OTU17) belonging to cluster 3b, four OTUs (OTU4, OTU6, OTU7, and OTU10) belonging to cluster 9, two OTUs (OTU3 and OTU11) belonging to cluster 2, and only one OTU (OTU9) belonging to cluster 4 (**Figure 3**).

**FIGURE 4:**
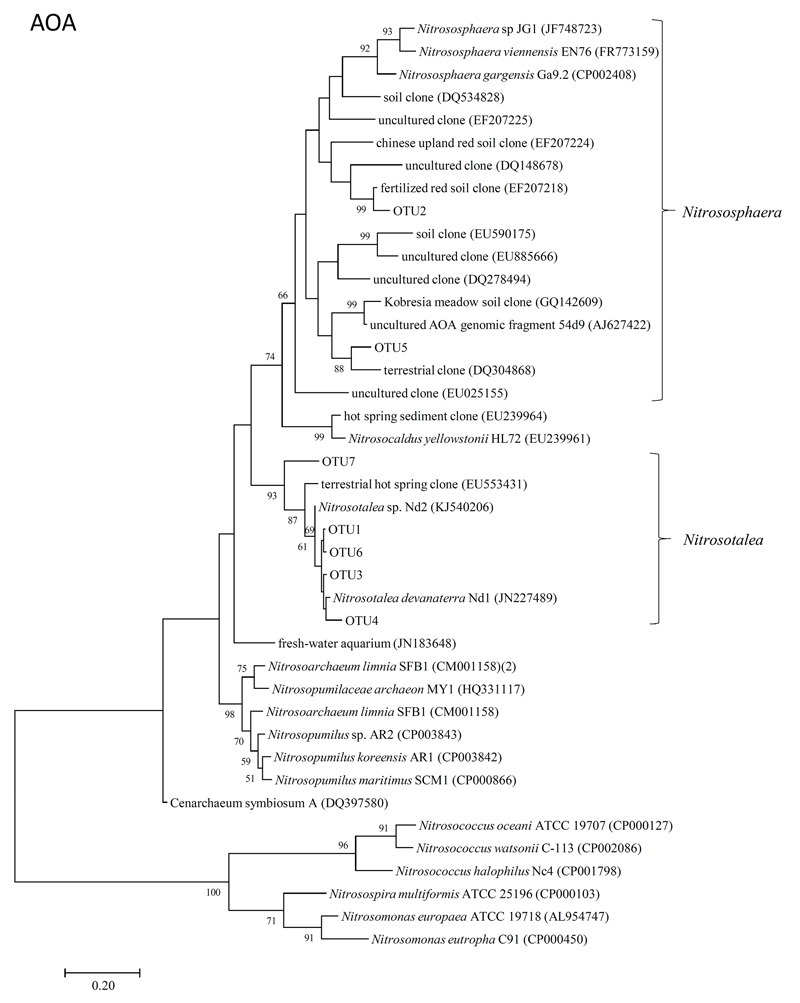
Neighbor-joining phylogenetic tree of archaeal *amoA* OTUs obtained in the present study. Bootstrap values (>50%) are indicated at branch points. The scale bar represents 10% estimated sequence divergence.

The representative sequences of seven dominant OTUs (>1.0% in all samples) of AOA *amoA* were used in the phylogenetic analysis. We used cluster and subcluster identification for the AOA *amoA* as previously defined (Pester et al., 2012). The AOA phylogenetic tree revealed that five dominant OTUs (OTU1, OTU3, OTU4, OTU6, and OTU7) were affiliated with *Nitrosotalea*, and two dominant OTUs (OTU2 and OTU5) were affiliated with *Nitrososphaera* (**Figure 4**).

### Community Structures of AOA and AOB

Alpha diversity indices of AOB in all treatments did not show significant alteration after incubation for 14 days (**Table 1**). For AOA, only the Control samples showed significantly altered alpha diversity indices after incubation for 14 days (**Table 1**). This suggested that some dominant OTUs of AOA might grow faster than others in the Control samples.

**Table 1:** *p*-values in *t*-tests comparing 0-day and 14-day samples in each treatment.

Genes	Treatment	Observed OTUs (OTU richness)	Estimated OTUs (Chao1 richness)	Inverse Simpson index	Shannon’s diversity index (H′)	Shannon’s species evenness (*E*)
AOB *amoA*	Control	0.140	0.523	0.683	0.649	0.841
	PHH	0.549	0.468	0.999	0.592	0.620
	DCD	0.405	0.250	0.149	0.254	0.289
AOA *amoA*	Control	**0.044**	0.650	**0.016**	**0.005**	**0.001**
	PHH	0.754	0.058	0.909	0.697	0.356
	DCD	0.827	0.945	0.572	0.825	0.956

*The *p*-values < 0.05 are in bold.*

**FIGURE 5:**
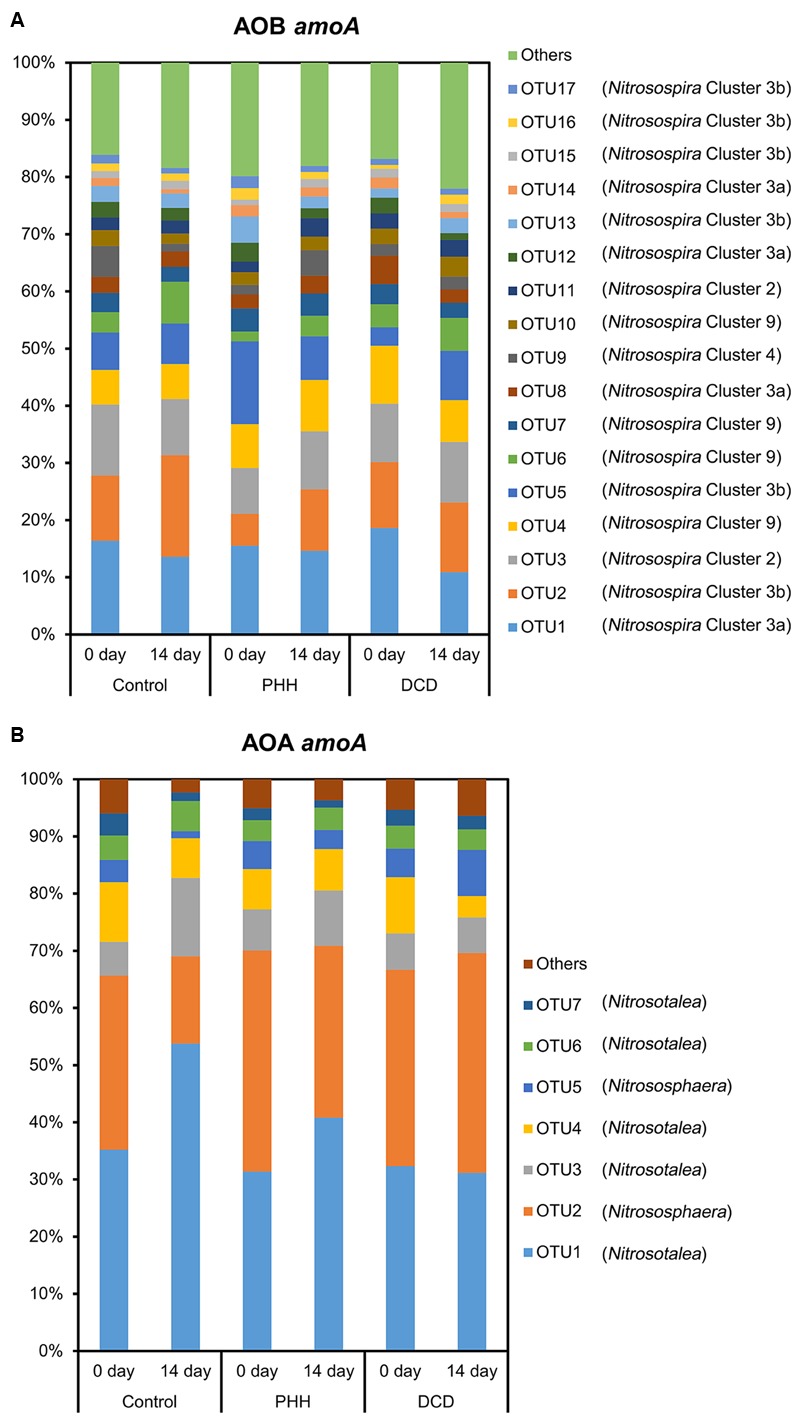
Relative abundance of dominant AOB *amoA* OTUs **(A)** and dominant AOA *amoA* OTUs **(B)** in the soils. Control, soil treated with ammonium sulfate [(NH_4_)_2_SO_4_]; PHH, soils treated with (NH_4_)_2_SO_4_ + PHH; DCD, (NH_4_)_2_SO_4_ + DCD.

The average relative abundance of the 17 dominant OTUs (>1.0% in all samples) of AOB *amoA* in triplicate samples from the individual groups showed few changes. OTU1, OTU2, OTU3, OTU4, and OTU5 accounted for approximately 50% of the sequence reads in all soil samples (**Figure 5A**). ANOSIM and unweighted UniFrac analyses showed that there was no significant difference in the community structure of AOB among all soil samples (Supplementary Tables S2, S3). The weighted UniFrac analysis showed marked difference not only between 0- and 14-day samples in all treatments but also between NI-amended soil samples and Control after incubation for 14 days (Supplementary Table S3).

Among *Nitrosospira* clusters, only cluster 4 in the Control and cluster 3a in the DCD amended soil showed significant decrease in their relative abundance (*p* = 0.046 for cluster 4 and *p* = 0.012 for cluster 3a in paired *t*-tests) after incubation for 14 days. Furthermore, the Metastats analyses indicated significant changes in all comparisons (**Figure 5A** and Supplementary Table S4). After incubation for 14 days, in the Control samples, OTU2 and OTU6, belonging to *Nitrosospira* cluster 3b and 9, showed increased relative abundance, whereas OTU1, OTU9, and OTU14, belonging to clusters 3a and 4, showed decreased relative abundance. In the treatment with PHH, only OTU2 showed an increased relative abundance after incubation for 14 days. In the treatment with DCD, OTU16, belonging to cluster 3b, showed increased relative abundance, whereas OTU1 and OTU8, belonging to cluster 3a, showed decreased relative abundance.

Comparing different treatments after incubation for 14 days showed that several OTUs had significantly different relative abundances (**Figure 5A** and Supplementary Table S4). In the treatment with PHH, the relative abundance of OTU2 and OTU6, belonging to *Nitrosospira* clusters 3b and 9, was lower than the abundance in the Control at day 14. The relative abundance of OTU9, belonging to *Nitrosospira* cluster 4, was higher than that in Control at day 14. In the treatment with DCD, only OTU2 showed a lower abundance than that in the Control at day 14.

Among the seven dominant OTUs (>1.0% in all samples) of AOA *amoA*, OTU1 and OTU2 accounted for over 65% of the sequence reads in all soil samples (**Figure 5B**). Unlike ANOSIM and unweighted UniFrac analyses, weighted UniFrac analysis indicated a significant difference in the community structure of AOA among all soil samples (Supplementary Tables S2, S3). *Nitrosotalea* showed a significant increase (*p* = 0.003 in a paired *t*-test) whereas *Nitrososphaera* showed a significant decrease (*p* = 0.006 in a paired *t*-test) in their relative abundance in the Control after incubation for 14 days. Metastats analyses revealed a significantly changed abundance of each of the seven dominant AOA *amoA* OTUs in the Control samples after incubation for 14 days (Supplementary Table S5). Among them, OTU1, OTU3, and OTU6, belonging to the *Nitrosotalea* cluster, showed increased relative abundance, whereas OTU2 and OTU5, belonging to the *Nitrososphaera* cluster, as well as OTU4 and OTU7 (belonging to the *Nitrosotalea* cluster), showed decreased relative abundance (**Figure 5B** and Supplementary Table S5).

Although the weighted UniFrac analysis indicated significant changes in AOA community structures in the soil samples incubated with PHH or DCD for 14 days, the UniFrac distance values were rather low (Supplementary Table S3). In addition, the Metastats analysis did not identify any OTU with significant changes in its abundance in these samples (Supplementary Table S5). Thus, neither PHH nor DCD had a significant effect on the community structure of AOA.

Comparing the different treatments after incubation for 14 days, three (in the treatment with PHH) and six (in the treatment with DCD) OTUs of AOA *amoA* showed significant differences in their relative abundances. In the PHH treatment, higher abundances were observed in OTU2 and OTU5, and a lower abundance was determined in OTU6, compared with those in the Control samples (**Figure 5B** and Supplementary Table S5). In the DCD treatment, the relative abundances of OTU1, OTU3, OTU4, and OTU6, belonging to the *Nitrosotalea* cluster, were lower than in the Control samples at day 14 (Supplementary Table S5). In contrast, the relative abundances of OTU2 and OTU5, belonging to the *Nitrososphaera* cluster, were higher than in the Control samples (**Figure 5B** and Supplementary Table S5).

**FIGURE 6:**
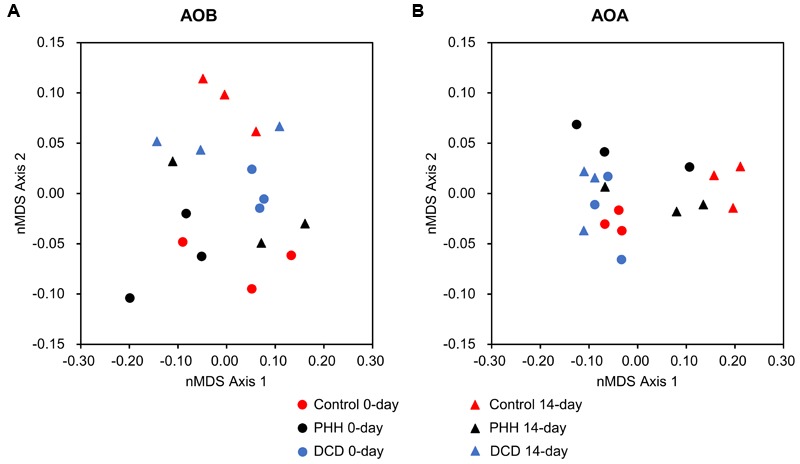
Non-metric multidimensional scaling plots of Hellinger-transformed Bray–Curtis dissimilarity matrices describing microbial communities of AOB **(A)** and AOA **(B)**.

Beta-diversities of AOB and AOA were presented as nMDS plots (**Figure 6**), which were in accord with the above analyses.

## Discussion

In the preliminary experiment, the level of ${\mathrm{NO}}_{3}^{–}$–N increased with the increase in the amended PHH concentration in 0-day soil samples (Supplementary Figure S1B), suggesting a contribution of PHH to the determined level of ${\mathrm{NO}}_{3}^{–}$–N. The concentration of PHH used for subsequent experiments was low (10 mmol⋅kg^-1^ dry soil); therefore, the amended PHH should not affect the measurement of ${\mathrm{NO}}_{3}^{–}$–N in the soil.

${\mathrm{NH}}_{4}^{+}$ is converted to ${\mathrm{NO}}_{3}^{–}$ via ${\mathrm{NO}}_{2}^{–}$ by nitrifying bacteria, leading to a decline in the ${\mathrm{NH}}_{4}^{+}$ concentration and an increase in the ${\mathrm{NO}}_{3}^{–}$ concentration in the soil. The addition of DCD and PHH resulted in an effective inhibition of ${\mathrm{NO}}_{3}^{–}$–N production in the Andosols (by 62.1 and 62.5% on day 3, and by 70.3 and 75.4% on day 14, respectively) (**Figure 1B**).

Dynamics of AOA and AOB *amoA* abundance in soil microcosms treated with NIs were determined. Consistent with a previous study (Kowalchuk and Stephen, 2001), the abundance of AOB *amoA* in the Control was increased by the addition of (NH_4_)_2_SO_4_ at 280 mg ${\mathrm{NH}}_{4}^{+}$–N kg^-1^ soil, with a 2.8-fold increase in the gene copy number (**Figure 2A**). Notably, the abundance of AOA *amoA* in the Control was stimulated by such high ${\mathrm{NH}}_{4}^{+}$ concentration, with an approximately 3.4-fold increase in the gene copy number (**Figure 2B**). AOA growth is favored in low N status soils (Nicol et al., 2008; Di et al., 2009, 2010a; Zhang et al., 2012), and its population could be potentially inhibited by the application of animal urine (Di et al., 2009, 2010a; Erguder et al., 2009). Daebeler et al. (2015) reported that AOA growth was significantly stimulated by low level of ${\mathrm{NH}}_{4}^{+}$ (48 mg ${\mathrm{NH}}_{4}^{+}$–N kg^-1^ soil); however, the growth was inhibited at higher levels (480 mg ${\mathrm{NH}}_{4}^{+}$–N kg^-1^ soil). The increasing abundance of AOA *amoA* was also observed in treatments with granulated ammonium sulfate nitrate (ASN) fertilizer (18.5% ${\mathrm{NH}}_{4}^{+}$–N, 7.5% ${\mathrm{NO}}_{3}^{–}$–N, and 13% S) (Kleineidam et al., 2011). It is possible that the increasing copy number of AOA *amoA* observed in our study could be attributed to three OTUs (OTU1, OTU3, and OTU6) affiliated with *Nitrosotalea* (**Figures 4, 5B**), which accounted for approximately 50% of the sequence reads in all soil samples. Previous studies showed that the acidophilic thaumarchaeal ammonia oxidizer could grow in media containing total ${\mathrm{NH}}_{4}^{+}$ concentrations as high as 10 mM (Lehtovirta-Morley et al., 2011). Thus, low pH (pH = 5.4) and high level of ${\mathrm{NH}}_{4}^{+}$ in this Andosol soil might favor rapid growth of AOA strains belonging to *Nitrosotalea*.

Compared with the Control samples, a significantly lower abundance of AOB *amoA* was observed in the presence of DCD and PHH from days 6 to 14 (**Figure 2A**), indicating similar dynamics between the AOB population and the net nitrification rate (Aarnio and Martikainen, 1992; Patra et al., 2006; Schauss et al., 2009; Di and Cameron, 2011; Wu et al., 2012; Liu et al., 2014). The inhibitory effect of PHH and DCD on AOA was similar to that observed in AOB. Although some studies suggested that AOA and AOB exhibited distinctly different responses to inhibitors because of certain essential differences in their physiology, biochemistry, and genetics (Schauss et al., 2009; Kleineidam et al., 2011), in the present study, both DCD and PHH showed strong inhibitory effects not only on AOB but also on AOA. PHH inhibited the growth of AOB through inhibiting the activity of the HAO enzyme in bacteria, however, it is unclear how PHH inhibited the growth of AOA because there is no homolog of the AOB *hao* gene in AOA genomes. Thus, the inhibition mechanism of PHH on the growth of AOA or on ammonia oxidation should be explored in the future.

The phylogenetic tree of AOB *amoA* revealed that all the 17 dominant OTUs of bacterial *amoA* sequences in the Andosols were exclusively within the *Nitrosospira* lineage (**Figure 3**), the dominant genus in many agricultural field soils (Kowalchuk and Stephen, 2001). Ten OTUs grouped into cluster 3, which appeared to be widely distributed in all kinds of soils and was associated with high concentrations of ${\mathrm{NH}}_{4}^{+}$ (Avrahami et al., 2003). Four OTUs grouped into cluster 9, which might be characteristic of soils affected by field management (Avrahami and Conrad, 2003) and of irrigated agriculture soils with low ${\mathrm{NH}}_{4}^{+}$ concentrations (Oved et al., 2001). OTU3 and OTU11 grouped into cluster 2, and OTU9 grouped into cluster 4. Cluster 4 may be characteristic for soils from the cold-temperate regions (Jiang and Bakken, 1999), and cluster 2 has been observed frequently in acidic agricultural soils (Stephen et al., 1998; Kowalchuk et al., 2000; Laverman et al., 2001). The OTUs affiliated with the cluster 3b (OTU2 in the Control and PHH samples, OTU16 in the DCD samples) showed increased abundance after incubation for 14 days; whereas, those affiliated with the cluster 3a (OTU1 and OTU14 in the Control samples, OTU1 and OTU8 in the DCD samples) showed decreased abundance. This suggested that the *Nitrosospira* clusters 3a and 3b responded in different ways to high concentrations of ammonium. Except for the *Nitrosospira* cluster 3, the other clusters did not show significant change in all treatments, suggesting less sensitivity to the treatment. Variations in AOB OTU abundance between NIs and the Control samples after 14-day incubation suggested varied sensitivity of AOB OTUs to different NIs.

In the present study, two dominant OTUs (OTU2 and OTU5) of AOA *amoA* were classified into the *Nitrososphaera* cluster (**Figure 4**), which was detected in various environments, including soil, in previous diversity studies on soil archaeal *amoA* (Francis et al., 2005; Leininger et al., 2006; Nicol et al., 2008; Wessén et al., 2011). Five other dominant OTUs (OTU1, OTU3, OTU4, OTU6, and OTU7), which accounted for approximately 60% of the sequence reads in all soil samples, were grouped into the *Nitrosotalea* cluster (**Figure 4**), which is dominant in acidic arable soil as acidophilic AOAs (Lehtovirta-Morley et al., 2011). The OTUs affiliated with *Nitrosotalea* (OTU1, OTU3, and OTU6) showed increased abundance in the Control samples after incubation for 14 days, whereas the others (OTU2 and OTU5, which were affiliated with *Nitrososphaera*; OTU4 and OTU7, which were affiliated with *Nitrosotalea*) showed decreased abundance. This suggested that not all of the *Nitrosotalea*, which are acidophilic *Archaea* nitrifiers, could grow well in an acidic soil with a high concentration of ammonium, and *Nitrososphaera*, which are ubiquitously distributed *Archaea* nitrifiers, could not grow well under this condition. Both NIs had no significant effect on the community structure of AOA; therefore, variations in AOA OTU abundance between NIs and the Control samples after 14-day incubation were mainly caused by growth of dominant AOA OTUs in the Control samples.

Dicyandiamide, which shows a bacteriostatic effect, as demonstrated by the reversibility of growth inhibition, differs greatly from PHH in its mechanism of inhibition (Zacherl and Amberger, 1990). Our results suggested that DCD inhibited the growth of AOA and ammonium oxidation by AOA in acidic soil, which is consistent with previous reports (Lehtovirta-Morley et al., 2011, 2013; Zhang et al., 2012).

Phenylhydrazine hydrochloride, which is a potent carbonyl reagent, inhibits several enzymes, such as alanine racemase (Nomura et al., 2001), catechol oxidases (Lerner et al., 1971), and enacyloxin oxidase (Oyama et al., 1994); therefore, it might be toxic to plants and microbes other than nitrifiers. This might restrict the use of PHH in agricultural practice. Thus, organohydrazine-based NIs targeting HAO with lower toxicity to microbes and plants should be developed in the future. For this reason, the analyses in the present study did not cover the overall microbial community, which should be done after an analog of PHH with lower toxicity is identified in the future.

Phenylhydrazine hydrochloride only inhibit the activity of HAO but not AMO; therefore, the addition of PHH into soil will cause transient accumulation of NH_2_OH, the substrate of HAO. The chemical decomposition of hydroxylamine produces N_2_O (Bremner et al., 1980); however, such emission of N_2_O could occur for a relatively short period of time until the microbial cells suffer from starvation because of shortage of energy. Thus, the quantity of N_2_O released from transiently accumulated NH_2_OH could be limited. To mitigate the emission of N_2_O, application of HAO inhibitors should be avoided in strongly acidic soil with high levels of manganese, which facilitates the chemical decomposition of NH_2_OH (Heil et al., 2016).

Although the PCR primers we used could detect the *amoA* gene in AOB and AOA, they did not match the sequences of the recently reported *amoA* gene in complete ammonia oxidization (Comammox) microorganisms (Daims et al., 2015; van Kessel et al., 2015). Thus, it is possible that AOB and AOA only partially contributed to the nitrification activity in the soil microcosm. For this reason, detection of the *amoA* gene in Comammox microorganisms will help us to better understand the mechanism underlying the inhibition of nitrification by NIs.

## Conclusion

Our results revealed that nitrification in agricultural Andosols was stimulated by (NH_4_)_2_SO_4_ addition but was almost completely inhibited by PHH and DCD based on the changes in ${\mathrm{NO}}_{3}^{–}$–N concentration and variation in the AOB and AOA abundance. Both PHH and DCD had limited effect on the community structure of AOB and no effect on the community structure of AOA. Based on our observation and a previous report (Wu et al., 2012), PHH appears to have toxic effects on archaea although archaea lack *hao*; thus, further studies are required to understand the mechanism underlying this phenomenon and to develop organohydrazine-based NIs targeting HAO.

## Author Contributions

WY, YW, and MH conceived and designed the study. WY, YW, KT, and ST acquired the data. WY and YW analyzed the data. WY, YW, KT, ST, and MH drafted and critically evaluated the manuscript. All authors approved the final version of the manuscript.

## Conflict of Interest Statement

The authors declare that the research was conducted in the absence of any commercial or financial relationships that could be construed as a potential conflict of interest.

## Footnotes

1 http://fungene.cme.msu.edu/FunGenePipeline/framebot/form.spr

2 https://www.r-project.org/
