# Supplementary material for: Comparison of the Effects of Phenylhydrazine Hydrochloride and Dicyandiamide on Ammonia-Oxidizing Bacteria and Archaea in Andosols
Source: Front Microbiol. 2017 Nov 14;8:2226. doi: 10.3389/fmicb.2017.02226 (PMC5694480; doi:10.3389/fmicb.2017.02226)
Supplement: Supplementary file 1 [file Table_1.DOCX]

**Table S1 |** Summary of the alpha diversity indices of ammonia-oxidizing bacteria (AOB) and ammonia-oxidizing archaea (AOA) in soil samples

| Genes | Treatment | No. of filtered sequences | No. of sequences for analysis | Good's library coverage | No. of OTUs observed (OTU richness) | No. of OTUs estimated (Chao1 richness) | Inverse Simpson index | Shannon's diversity index (H') | Shannon's species evenness (E) |
| --- | --- | --- | --- | --- | --- | --- | --- | --- | --- |
| AOB *amoA* | Control-0day-1 | 4023 | 1825 | 98.5% | 86 | 145 | 15.50 | 3.27 | 0.73 |
|  | Control -0day-2 | 5754 | 1825 | 98.4% | 88 | 142 | 11.65 | 3.03 | 0.68 |
|  | Control -0day-3 | 7575 | 1825 | 98.5% | 89 | 110 | 12.08 | 3.06 | 0.68 |
|  | Control -14day-1 | 5458 | 1825 | 98.1% | 97 | 209 | 13.34 | 3.23 | 0.71 |
|  | Control -14day-2 | 4083 | 1825 | 98.7% | 92 | 108 | 12.62 | 3.18 | 0.70 |
|  | Control -14day-3 | 4975 | 1825 | 98.1% | 92 | 148 | 11.98 | 3.06 | 0.68 |
|  | PHH-0day-1 | 4988 | 1825 | 98.1% | 99 | 142 | 14.31 | 3.27 | 0.71 |
|  | PHH-0day-2 | 1825 | 1825 | 98.7% | 86 | 109 | 13.21 | 3.17 | 0.71 |
|  | PHH-0day-3 | 4660 | 1825 | 98.3% | 86 | 138 | 13.13 | 3.17 | 0.71 |
|  | PHH-14day-1 | 4943 | 1825 | 98.4% | 98 | 127 | 15.21 | 3.34 | 0.73 |
|  | PHH-14day-2 | 9042 | 1825 | 98.5% | 80 | 109 | 11.49 | 2.96 | 0.68 |
|  | PHH-14day-3 | 4352 | 1825 | 98.4% | 88 | 139 | 13.95 | 3.16 | 0.71 |
|  | DCD-0day-1 | 6214 | 1825 | 98.1% | 92 | 158 | 12.51 | 3.12 | 0.69 |
|  | DCD-0day-2 | 6103 | 1825 | 98.6% | 86 | 140 | 12.39 | 3.12 | 0.70 |
|  | DCD-0day-3 | 5583 | 1825 | 98.0% | 98 | 154 | 12.39 | 3.13 | 0.68 |
|  | DCD-14day-1 | 5861 | 1825 | 97.9% | 90 | 168 | 12.96 | 3.08 | 0.68 |
|  | DCD-14day-2 | 2522 | 1825 | 97.8% | 109 | 184 | 17.43 | 3.42 | 0.73 |
|  | DCD-14day-3 | 3399 | 1825 | 98.0% | 101 | 158 | 16.70 | 3.41 | 0.74 |
|  |  |  |  |  |  |  |  |  |  |
| AOA *amoA* | Control-0day-1 | 5529 | 3719 | 99.5% | 45 | 64 | 3.94 | 1.81 | 0.47 |
|  | Control-0day-2 | 7772 | 3719 | 99.5% | 43 | 81 | 4.48 | 1.90 | 0.51 |
|  | Control-0day-3 | 4025 | 3719 | 99.5% | 47 | 64 | 4.26 | 1.85 | 0.48 |
|  | Control-14day-1 | 5129 | 3719 | 99.5% | 39 | 66 | 2.98 | 1.50 | 0.41 |
|  | Control-14day-2 | 4511 | 3719 | 99.5% | 34 | 68 | 3.09 | 1.53 | 0.43 |
|  | Control-14day-3 | 5259 | 3719 | 99.5% | 34 | 97 | 2.77 | 1.45 | 0.41 |
|  | PHH-0day-1 | 6786 | 3719 | 99.6% | 40 | 58 | 3.83 | 1.79 | 0.49 |
|  | PHH-0day-2 | 6414 | 3719 | 99.6% | 37 | 54 | 3.50 | 1.61 | 0.45 |
|  | PHH-0day-3 | 4667 | 3719 | 99.7% | 39 | 47 | 3.18 | 1.67 | 0.46 |
|  | PHH-14day-1 | 4231 | 3719 | 99.5% | 38 | 72 | 3.58 | 1.62 | 0.45 |
|  | PHH-14day-2 | 4885 | 3719 | 99.4% | 50 | 79 | 3.95 | 1.80 | 0.46 |
|  | PHH-14day-3 | 3719 | 3719 | 99.6% | 34 | 58 | 3.07 | 1.49 | 0.42 |
|  | DCD-0day-1 | 4404 | 3719 | 99.4% | 50 | 82 | 4.21 | 1.85 | 0.47 |
|  | DCD-0day-2 | 4267 | 3719 | 99.5% | 44 | 87 | 4.22 | 1.80 | 0.48 |
|  | DCD-0day-3 | 4743 | 3719 | 99.7% | 37 | 57 | 3.86 | 1.75 | 0.49 |
|  | DCD-14day-1 | 4966 | 3719 | 99.7% | 36 | 44 | 3.85 | 1.76 | 0.49 |
|  | DCD-14day-2 | 5823 | 3719 | 99.5% | 44 | 95 | 3.46 | 1.68 | 0.44 |
|  | DCD-14day-3 | 5845 | 3719 | 99.5% | 46 | 91 | 4.28 | 1.90 | 0.50 |
