# Supplementary material for: Comparison of the Effects of Phenylhydrazine Hydrochloride and Dicyandiamide on Ammonia-Oxidizing Bacteria and Archaea in Andosols
Source: Front Microbiol. 2017 Nov 14;8:2226. doi: 10.3389/fmicb.2017.02226 (PMC5694480; doi:10.3389/fmicb.2017.02226)
Supplement: Supplementary file 2 [file Table_2.DOCX]

**Table S2 |** ANOSIM analysis of community structures of ammonia-oxidizing bacteria (AOB) and ammonia-oxidizing archaea (AOA)

|  | Comparison | R value | *p* value |
| --- | --- | --- | --- |
| AOB | Control-0day vs. Control-14day | 0.48 | 0.09 |
|  | PHH-0day vs. PHH-14day | 0.11 | 0.30 |
|  | DCD-0day vs. DCD-14day | 0.67 | 0.10 |
|  |  |  |  |
|  | PHH-14day vs. Control-14day | 0.37 | 0.10 |
|  | DCD-14day vs. Control-14day | 0.33 | 0.20 |
|  |  |  |  |
| AOA | Control-0day vs. Control-14day | 1.00 | 0.10 |
|  | PHH-0day vs. PHH-14day | -0.11 | 0.60 |
|  | DCD-0day vs. DCD-14day | 0.04 | 0.39 |
|  |  |  |  |
|  | PHH-14day vs. Control-14day | 0.44 | 0.09 |
|  | DCD-14day vs. Control-14day | 1.00 | 0.10 |
