# Supplementary material for: Comparison of the Effects of Phenylhydrazine Hydrochloride and Dicyandiamide on Ammonia-Oxidizing Bacteria and Archaea in Andosols
Source: Front Microbiol. 2017 Nov 14;8:2226. doi: 10.3389/fmicb.2017.02226 (PMC5694480; doi:10.3389/fmicb.2017.02226)
Supplement: Supplementary file 3 [file Table_3.DOCX]

**Table S3 |** UniFrac analyses of ammonia-oxidizing archaea (AOA) and ammonia-oxidizing bacteria (AOB) community structures

|  | Groups | Unweighted | |  | Weighted | |
| --- | --- | --- | --- | --- | --- | --- |
|  |  | UniFrac distance | *p* |  | UniFrac distance | *p* |
| AOB | Control-0day vs. Control-14day | 0.94 | 0.417 |  | 0.86 | **<0.0010** |
|  | PHH-0day vs. PHH-14day | 0.76 | 0.422 |  | 0.69 | **<0.0010** |
|  | DCD-0day vs. DCD-14day | 0.94 | 0.486 |  | 0.86 | **<0.0010** |
|  | PHH-14day vs. Control-14day | 0.94 | 0.416 |  | 0.86 | **<0.0010** |
|  | DCD-14day vs. Control-14day | 0.78 | 0.309 |  | 0.63 | **<0.0010** |
|  |  |  |  |  |  |  |
| AOA | Control-0day vs. Control-14day | 1.00 | 0.536 |  | 1.00 | **<0.0010** |
|  | PHH-0day vs. PHH-14day | 0.21 | 1.000 |  | 0.36 | **<0.0010** |
|  | DCD-0day vs. DCD-14day | 0.56 | 1.000 |  | 0.37 | **<0.0010** |
|  | PHH-14day vs. Control-14day | 0.60 | 0.712 |  | 0.42 | **<0.0010** |
|  | DCD-14day vs. Control-14day | 1.00 | 0.580 |  | 1.00 | **<0.0010** |

The *p* values < 0.05 are in bold.
