# Supplementary material for: Comparison of the Effects of Phenylhydrazine Hydrochloride and Dicyandiamide on Ammonia-Oxidizing Bacteria and Archaea in Andosols
Source: Front Microbiol. 2017 Nov 14;8:2226. doi: 10.3389/fmicb.2017.02226 (PMC5694480; doi:10.3389/fmicb.2017.02226)
Supplement: Supplementary file 4 [file Table_4.DOCX]

**Table S4 |** *p*-values in Metastats analyses of ammonia-oxidizing bacteria (AOB) *amoA*

| OTUs | Control-0day vs.  Control-14day | PHH-0day vs.  PHH-14day | DCD-0day vs.  DCD-14day | PHH-14day vs.  Control-14day | DCD-14day vs.  Control-14day |
| --- | --- | --- | --- | --- | --- |
| OTU1 | **0.046** | 0.842 | **0.005** | 0.755 | 0.075 |
| OTU2 | **0.010** | **0.032** | 0.816 | **0.001** | **0.030** |
| OTU3 | 0.354 | 0.257 | 0.734 | 0.881 | 0.559 |
| OTU4 | 0.949 | 0.547 | 0.174 | 0.057 | 0.448 |
| OTU5 | 0.891 | 0.269 | 0.183 | 0.916 | 0.690 |
| OTU6 | **0.003** | 0.158 | 0.417 | **0.028** | 0.441 |
| OTU7 | 0.296 | 0.875 | 0.324 | 0.163 | 0.913 |
| OTU8 | 0.865 | 0.508 | **0.006** | 0.518 | 0.439 |
| OTU9 | **0.032** | 0.069 | 0.719 | **0.034** | 0.207 |
| OTU10 | 0.091 | 0.880 | 0.611 | 0.538 | 0.177 |
| OTU11 | 0.924 | 0.071 | 0.363 | 0.202 | 0.212 |
| OTU12 | 0.497 | 0.094 | 0.090 | 0.521 | 0.058 |
| OTU13 | 0.872 | 0.153 | 0.260 | 0.753 | 0.919 |
| OTU14 | **0.006** | 0.761 | 0.071 | 0.181 | 0.072 |
| OTU15 | 0.648 | 0.290 | 0.413 | 0.932 | 0.646 |
| OTU16 | 0.949 | 0.431 | **0.039** | 0.929 | 0.587 |
| OTU17 | 0.478 | 0.215 | 0.946 | 0.919 | 0.867 |

The *p* values < 0.05 are in bold.
