# Supplementary material for: Comparison of the Effects of Phenylhydrazine Hydrochloride and Dicyandiamide on Ammonia-Oxidizing Bacteria and Archaea in Andosols
Source: Front Microbiol. 2017 Nov 14;8:2226. doi: 10.3389/fmicb.2017.02226 (PMC5694480; doi:10.3389/fmicb.2017.02226)
Supplement: Supplementary file 5 [file Table_5.DOCX]

**Table S5 |** *p*-values in Metastats analyses of ammonia-oxidizing archaea (AOA) *amoA*

| OTUs | Control-0day vs.  Control-14day | PHH-0day vs.  PHH-14day | DCD-0day vs.  DCD-14day | PHH-14day vs. Control-14day | DCD-14day vs. Control-14day |
| --- | --- | --- | --- | --- | --- |
| OTU1 | **0.012** | 0.398 | 0.501 | 0.076 | **0.009** |
| OTU2 | **0.008** | 0.421 | 0.294 | **0.038** | **0.022** |
| OTU3 | **0.019** | 0.512 | 0.837 | 0.116 | **0.029** |
| OTU4 | **0.030** | 0.880 | 0.086 | 0.873 | **0.023** |
| OTU5 | **0.013** | 0.186 | 0.149 | **0.026** | **0.004** |
| OTU6 | **0.027** | 0.728 | 0.105 | **0.014** | **0.018** |
| OTU7 | **0.021** | 0.306 | 0.528 | 0.785 | 0.086 |

The *p* values < 0.05 are in bold.
