# Supplementary material for: Comparison of the Effects of Phenylhydrazine Hydrochloride and Dicyandiamide on Ammonia-Oxidizing Bacteria and Archaea in Andosols
Source: Front Microbiol. 2017 Nov 14;8:2226. doi: 10.3389/fmicb.2017.02226 (PMC5694480; doi:10.3389/fmicb.2017.02226)
Supplement: Supplementary file 6 [file Presentation_1.PDF]

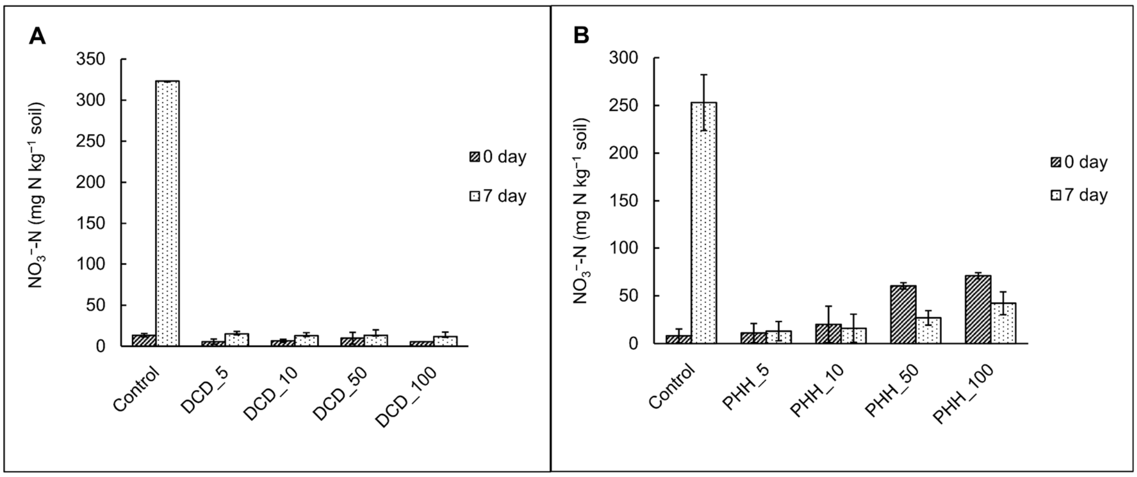

FIGURE S1 | Concentrations of nitrate nitrogen (NO_3^-–N) in the soil amended with DCD (A) and PHH (B). Control, soil treated with ammonium sulfate ($(NH_4)_2SO_4$); DCD, $(NH_4)_2SO_4$ + dicyandiamide (DCD); PHH, soils treated with $(NH_4)_2SO_4$ + phenylhydrazine hydrochloride (PHH). 5, 10, 50, and 100 indicate the concentrations (mmol·kg^{-1} dry soil) of DCD or PHH in soil. Error bars represent the standard deviation of means (SD) (n = 3).
